# Metatarsal fusion resisted bending as jerboas (Dipodidae) transitioned from quadrupedal to bipedal

**DOI:** 10.1098/rspb.2022.1322

**Published:** 2022-10-12

**Authors:** Carla Nathaly Villacís Núñez, Andrew P. Ray, Kimberly L. Cooper, Talia Y. Moore

**Affiliations:** ^1^ Mechanical Engineering Department, University of Michigan, Ann Arbor 48109, MI, USA; ^2^ Materials Science Engineering Department, University of Michigan, Ann Arbor 48109, MI, USA; ^3^ Robotics Department, Ecology and Evolutionary Biology Department, Museum of Zoology, University of Michigan, Ann Arbor 48109, MI, USA; ^4^ Department of Cell and Developmental Biology, University of California, San Diego 92093, CA, USA

**Keywords:** finite element, bone stress, fracture, CT scan, functional morphology

## Abstract

Hind limbs undergo dramatic changes in loading conditions during the transition from quadrupedal to bipedal locomotion. For example, the most early diverging bipedal jerboas (Rodentia: Dipodidae) are some of the smallest mammals in the world, with body masses that range between 2–4 g. The larger jerboa species exhibit developmental and evolutionary fusion of the central three metatarsals into a single cannon bone. We hypothesize that small body size and metatarsal fusion are mechanisms to maintain the safety factor of the hind limb bones despite the higher ground reaction forces associated with bipedal locomotion. Using finite-element analysis to model collisions between the substrate and the metatarsals, we found that body size reduction was insufficient to reduce bone stress on unfused metatarsals, based on the scaled dynamics of larger jerboas, and that fused bones developed lower stresses than unfused bones when all metatarsals are scaled to the same size and loading conditions. Based on these results, we conclude that fusion reinforces larger jerboa metatarsals against high ground reaction forces. Because smaller jerboas with unfused metatarsals develop higher peak stresses in response to loading conditions scaled from larger jerboas, we hypothesize that smaller jerboas use alternative dynamics of bipedal locomotion to reduce the impact of collisions between the foot and substrate.

## 1. Introduction

The transition from quadrupedal to facultative or obligate bipedal locomotion has convergently evolved in multiple mammal lineages, including apes and macropods at the largest sizes, moderately sized springhares and small rodents, such as kangaroo rats and jerboas. In each lineage, the change in support of the body weight, from four legs to two, increases loading on the hind limbs [1]. Furthermore, in contrast to quadrupedal locomotion, bipedal locomotion more frequently involves an aerial phase, especially in ‘ricochetal’ locomotion, which generates even greater ground reaction forces and loading on each hind limb [2,3]. The transition to bipedal locomotion therefore requires a complex suite of morphological changes to support these differences in both movement and loading [4].

Much of our understanding of the musculoskeletal strategies that compensate for the dynamical changes associated with the transition to bipedal locomotion have been limited to studies of large animals, whose economical, steady-state locomotion can be easily modelled and predicted [5,6]. Apes, dinosaurs (including birds) and large marsupials have diverse morphological adaptations to accommodate the changes in bone loading [7–9], probably reflecting different ancestral quadrupedal morphologies and different selective pressures that favoured the transition to bipedal locomotion from a variety of different quadrupedal morphologies. However, the extent to which previous research on large animals can be used to understand how smaller animals evolved bipedal locomotion is unclear.

For smaller animals, bipedal locomotion is frequently used in short bursts to increase the speed of an escape response [2,10]. While biomechanically advantageous traits are not necessarily the result of selection, predation is probably a strong selective force shaping the morphological traits that enable small prey to perform evasive locomotion. Facultatively bipedal lizards and hopping mice share similar morphological traits, including slight reduction of forelimb length, elongation of hind limb and elongation of the tail [11,12]. These morphological adaptations can be even more extreme in small, obligately bipedal animals that use short bursts of acceleration to evade predators. This is perhaps because an injury to a single limb is more likely to be detrimental to the fitness of an obligate, rather than facultative, biped.

Jerboas (family Dipodidae) are small, obligately bipedal hopping rodents whose ricochetal escape responses involve energetically costly manoeuvers, including unpredictable three-dimensional trajectories and vertical leaps of over 10 times hip height [13–15]. Single-leg peak ground-reaction forces in bipedal jerboas are over five times body weight [14], which is higher than in most large mammals [16]. The morphological divergence and diversity among jerboas has also evolved substantially to generate and withstand such explosive manoeuvers.

The superfamily Dipodoidea is the most taxonomically rich and oldest group of bipedal rodents, including 51 species, 33 of which are obligately bipedal jerboas [17]. The clade provides a rich resource for examining this biomechanical transition, including hind limb morphotypes that represent multiple combinations of morphological traits along the continuum between ancestral obligately quadrupedal to derived obligately bipedal forms [17].

**Figure 1.**
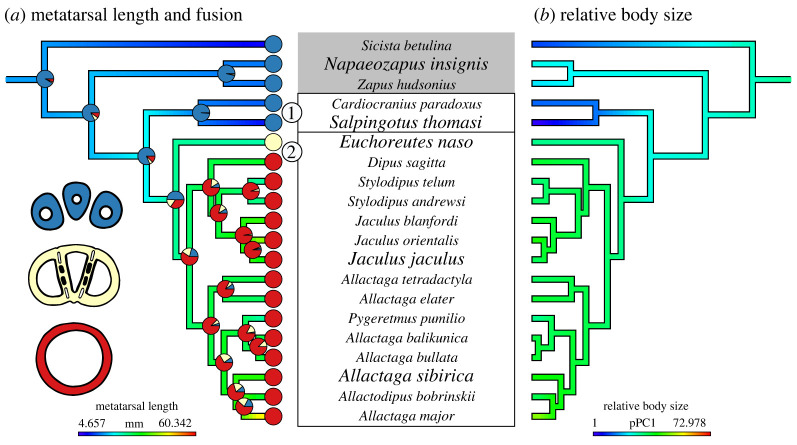
Ancestral state character mapping for species in Dipodoidea. In (*a*), the colours used to overlay the phylogenetic tree indicate the statistically reconstructed metatarsal length of common ancestors. The coloured pie charts on the tree indicate metatarsal fusion, corresponding to the diagram of cross-sectional shape on the left. In (*b*), the colours used to overlay the phylogenetic tree indicate the statistically reconstructed relative body size of common ancestors. The species names in a larger font size are included in the study and the species in the grey box are quadrupedal. That the earliest diverging obligately bipedal jerboas with unfused metatarsals (*Cardiocranius paradoxus*, *Salpingotus thomasi*, indicated in box 1) have similar metatarsal lengths (in blue on left), but greatly decrease body size (towards cooler colours on right) with respect to earlier diverging facultatively bipedal quadrupeds. Later diverging species (indicated in box 2) with at least partially fused metatarsals exhibit increases (towards warmer colours) in both metatarsal length and body size. Note: the ancestral state reconstruction was computed for a phylogenetic tree of 151 rodent species, then trimmed to only depict species in Dipodoidea, which is why the continuous traits (metatarsal length and relative body size) do not reach the maximum red values. Comparative methods and complete ancestral state reconstructions for all 151 species are included in the electronic supplementary material, S1. (Online version in colour.)

Obligately quadrupedal (*Sicista betulina*) and facultatively bipedal (*Napaeozapus insignis*) dipodoids are sister to all jerboas. These dipodoids have unfused metatarsals and are similar in body size to common mice (figure 1, grey box). The earliest diverging obligately bipedal jerboas are some of the smallest mammals in the world (*Salpingotus michaelis*, body mass ≈2–4 g) and are sister to all other jerboas (figure 1, box 1). A single extant jerboa species, *Euchoreutes naso* (figure 1, yellow tipped species in box 2), exhibits partially fused metatarsals and an intermediate body size (31 g) [18] and is sister to the subfamilies of three-toed (Dipodinae) and five-toed (Allactaginae) jerboas. In this species, the remnants of bone at the interfaces of adjacent metatarsals remain as columns that traverse the medullary cavity [19]. After complete metatarsal fusion in the last common ancestor of the three- and five-toed jerboas, body mass increased up to three orders of magnitude in the largest species of bipedal jerboa (*Allactaga major* body mass ≈400 g) (figure 1, all red tipped species in box 2) [20]. The apparent correlation in progression of body size and metatarsal fusion in jerboas suggests that these traits function to resist the greater ground reaction forces associated with bipedal locomotion.

Owing to the lack of complete jerboa fossils, we examine two potential scenarios that describe the relative timing of evolutionary changes in metatarsal fusion, body size and bipedal locomotion in jerboas. The first scenario is the ‘miniaturization bottleneck,’ which states that miniaturization was necessary in the common ancestor of all jerboas to evolve obligately bipedal locomotion, owing to the higher ground reaction forces associated with bipedal locomotion. Then metatarsal fusion subsequently reinforced the foot, thereby releasing the constraint and enabling evolutionary increases in body size. The second scenario considers metatarsal fusion and miniaturization to be ‘independent traits.’ In this scenario, the common ancestor of all jerboas would have evolved bipedal locomotion with unfused metatarsals and a medium body size. Then, independent, obligately bipedal lineages would have subsequently evolved either miniaturization or metatarsal fusion and an increase in body size. To determine which scenario is more likely, we can use phylogenetically informed statistical reconstruction of phenotypic traits to estimate the phenotypes of the common ancestor to all jerboas, based on extant data [21]. However, these estimates are not necessarily constrained by biomechanical feasibility. We will complement the phylogenetic estimates by modelling how size and shape interact to determine the strength of metatarsal bones.

In principle, metatarsal bones can be modelled as hollow tubes to test hypotheses regarding the mechanical function of fusion because the trabecular bone inside has much lower density. The flexural stiffness of a hollow tube in response to a deflection varies with respect to the second moment of area, which is proportional to $r_{\mathrm{outer}}^{4}-r_{\mathrm{inner}}^{4}$. Therefore, one fused bone with a large outer radius will bend less in response to a load than three bones with smaller radii sharing the same load, even if all bones have the same wall thickness. However, these simple calculations do not consider the overall three-dimensional shape of the bones or the complex inner structure of the *E. naso* metatarsus [22].

Finite-element analysis (FEA) can be used to compute the stresses developed in objects according to shape and loading conditions. Previous work to model the mechanical strength of biological structures using this approach has provided key insights into the selective pressures shaping their evolution [23–25]. Here, we use FEA to model bone stresses in five dipodoid species to gain insight into the morphological characteristics associated with the evolution of obligate bipedal locomotion in jerboas. Our study examines two hypotheses to determine how extant jerboas compensate for the higher forces associated with bipedal locomotion. This analysis sheds light on the biomechanical constraints that probably governed the evolution of bipedal locomotion in the extinct common ancestor of all jerboas:

— **hypothesis 1:** assuming that peak bipedal ground-reaction forces are proportional to body mass, less body mass with respect to foot length reduces relative bone loading such that unfused metatarsals can safely perform bipedal locomotion; and
— **hypothesis 2:** fusion reduces the magnitude of stresses developed in the metatarsals in response to the same loading conditions, given the same bone length and wall thickness.

The link between form and function in the foot bones is determined greatly by the impact of the bone with the substrate, which is a dynamic interaction. Dynamic FEA is commonly used to determine the medical consequences of collisions with humans [26,27], or to design bioinspired structures [28,29], but is rarely used in a comparative or evolutionary context. Here, we use dynamic models to determine how incorporating the dynamics of the collision between metatarsus and substrate and model complexity can affect the modelling results.

By comparing the stresses developed in metatarsal bones that vary in shape and size, we seek to provide insight into the biomechanical consequences of transitioning to bipedal locomotion in small terrestrial vertebrates. The results from our analyses provide insight into the coevolution of body size and metatarsal shape in bipedal desert rodents. The approach described here can also be used to understand the role of bone fusion in other animal limb elements.

## 2. Material and methods

### (a) Scanning specimens

Specimens were loaned from museum collections (details in the electronic supplementary material, table S2). We used skeletal measurements to compare the patterns of metatarsal length and body size evolution in Dipodoidea (figure 1; electronic supplementary material, methods). Each specimen was packed in floral foam and scanned with a Skyscan 1173 micro computed tomography (μCT) scanner (Bruker μCT, Kontich, Belgium). Specimens were scanned with 70 kV and 114 μA for all specimens except for the adult *Jaculus jaculus*, which was scanned at 60 kV and 113 μA. Specimens were scanned at resolutions resulting in 16.34 μm (*J. jaculus*), 14.92 μm (*Salpingotus thomasi*), 22.03 μm (*E. naso*), 16.34 μm (*N. insignis*) and 20.02 μm (*Allactaga sibirica*) camera pixel sizes.

Section images were reconstructed with the program NRecon (Bruker, Kontich, Belgium) or 3D Slicer [30] and exported as three-dimensional surface models (STL format). MeshLab [31] or 3D Slicer were used to segment out the three central metatarsal bones or the fused equivalent of the same bones. Unfused metatarsals were segmented individually.

Internal microstructures and protrusions were also eliminated to reduce the complexity of the model and reduce computation time.

### (b) Overview of finite-element modelling

We used two model conditions to test each specimen (details in table 1). In the first models, the bones are kept at their true scale, but a 3 mm displacement is scaled (listed in the electronic supplementary material, table S2) to their body size Previous FEA-based studies used estimates of body mass as proxies for scaling loading conditions [23,25,32]. Comparisons among species in these simulations demonstrate how the unscaled animal morphologies would respond to the scaled loading conditions associated with the bipedal ground-reaction forces measured in *J. jaculus* [13], and address hypothesis 1.

**Table 1.** Description of the models performed in this study with boundary conditions. (Scale refers to both the length and the cortical thickness; the scaled conditions are set to match *J. jaculus*. Displacement refers to the magnitude of displacement of the plate applied to the distal epiphysis of the metatarsus. The displacement values for the unscaled models are provided in the electronic supplementary material, table S3. See figure 2 for a visual representation of the boundary conditions and scaling.)

model	type	distribution	scale	displacement
1	dynamic	multiple node	actual size	scaled by geometric mean
2	dynamic	multiple node	scaled to *J. jaculus* size	3 mm

The second models scaled all other specimens to match the length of the *J. jaculus* metatarsus and used the same 3 mm displacement. Comparisons among species in these simulations provide insight into the bone stresses *J. jaculus* might experience if they had the metatarsal morphologies of their close relatives, and address hypothesis 2. By comparing the response of each species in scaled and unscaled simulations, we can determine whether reducing body size sufficiently reduces the ground-reaction forces to compensate for the structural weaknesses of the metatarsal shape. Similar comparisons between scaled and unscaled models have provided insight into the effect of size on bone function [24,32–34].

### (c) Scaling cortical thickness

The species in this dataset differ significantly in bone cortical thickness (electronic supplementary material, table S2), which interacts with metatarsal fusion to determine the strength of the bones. Bone cortical thickness scales with size among vertebrate animals, but lineage-specific and locomotion-specific factors determine the exact scaling relationship [35]. Although this study examines species in the same superfamily (Dipodoidea), the dataset spans multiple modes of locomotion and two orders of magnitude in body size, making it infeasible to apply any pre-existing scaling relationships to correct for the effect of body size in our comparisons. Instead, we scaled all specimens in the second models to match the cortical thickness of *J. jaculus*. We did this by first isometrically scaling both the inner and outer surfaces of the models to match the metatarsal lengths to that of *J. jaculus* (reported in the electronic supplementary material, table S2). Then, we scaled only the inner surfaces in the transverse direction (not adjusting length) to match the cortical thickness of *J. jaculus*. The centring point was adjusted to ensure that cortical thickness was relatively consistent throughout the length of the bone. For each species, the thickness scaling factor was calculated by using the relative ratio of the unscaled cortical thickness at midshaft to that of *J. jaculus*. The cortical thicknesses and lengths of the first models remained unaltered. Model creation and simplification are described more thoroughly in the electronic supplementary material, S2. The same *J. jaculus* model was used for both modelling conditions because it did not need to be scaled. Detailed methods regarding modelling the material properties of bone are included in the electronic supplementary material, S4.

### (d) Displacement conditions

The scaled plate displacement was set to 3 mm. This represents half of the vertical distance (6 mm) that the proximal portions of the metatarsals displaced while the distal portions of the metatarsals were in contact with the substrate, which we determined from previously collected fluoroscopic video [36]. However, preliminary models using the 6 mm displacement exhibited ground reaction forces that exceeded the values obtained empirically (4 N), probably owing to the constraint in rotation. Therefore, we decreased the displacement by half. In the unscaled models, the 3 mm displacements were scaled to body size. Because linear distances scale with volume and mass to the 1/3 power, we used the geometric mean of all linear limb element measurements to scale the displacements (electronic supplementary material, figure S1). See the electronic supplementary material, S1 for detailed methods regarding the linear limb measurements.

**Figure 2.**
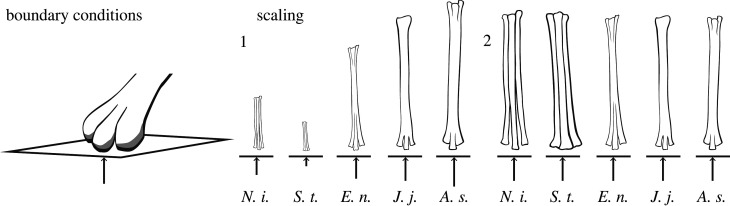
Diagram of the boundary conditions and scaling of the simulations, as described in table 1. For boundary conditions, both models use a solid plane to apply a dynamic displacement (denoted by the arrow) to a region of nodes in the bone mesh. For scaling, the length of the vertical arrow below each metatarsus depicts the vertical displacement for each simulation.

Both scaled and unscaled plate velocity was set at 1 m s^−1^. This was computed by dividing the displacement by the total time the foot was in contact with the substrate, also determined from fluoroscopic video. Because overall displacement was scaled to body size, the total duration of displacement varied with each unscaled model (electronic supplementary material, table S3). Based on the touchdown angle from the fluoroscopic videos of *J. jaculus*, the bone was oriented at a $45^{\circ }$ angle to the loading direction (figure 2*a*).

### (e) Dynamic finite-element analysis

We used LS-Dyna (Livermore Software Technology, Livermore, CA, USA) to simulate the dynamic collision between bone and substrate as a jerboa jumps on the ground. To achieve this condition, the proximal bone end epiphysis was fixed while the distal epiphysis collided with a rigid plate model. Shell elements of size 2.000 mm by 2.143 mm were chosen for this plate. We ensured that the maximum ground reaction force produced by each model would not exceed 4 N, which is the maximum ground reaction force recorded from *J. jaculus* [13].

Both the plate and the metatarsal models were integrated into a single file, in which the bones were oriented at 45° from the horizontal plate (figure 2*b*). The distal epiphyses of the bones were separated 1 mm from the shell plate. The proximal epiphyses of the bones were constrained in six degrees of freedom (displacement and rotation). K extension files were created in Hypermesh and submitted to the Great Lakes High-Performance Computing Cluster. Results were visualized in LS-PrePost (Livermore Software Technology, Livermore, CA, USA).

## 3. Results

Ancestral state reconstruction of metatarsal length, metatarsal fusion and relative body size in Dipodoidea provides context for different modelling conditions. Early diverging obligately bipedal pygmy jerboas, *C. paradoxus* and *Sa. thomasi* (figure 1, box 1), increase metatarsal length relative to body size, despite having metatarsals that are similar in size to those of closely related facultatively bipedal dipodoids, by greatly decreasing body size with respect to all sister taxa and ancestors. Ancestral state reconstruction suggests that pygmy jerboas may have reduced absolute metatarsal length with respect to their most recent common ancestors. In later diverging jerboas, the reconstructions support a single origin of metatarsal fusion and a consistently larger body size than earlier diverging dipodoids (figure 1, box 2). These phylogenetic patterns of morphological traits support using unscaled models to understand the early transition to bipedal locomotion and scaled models to understand the later diversification among bipedal species.

**Figure 3.**
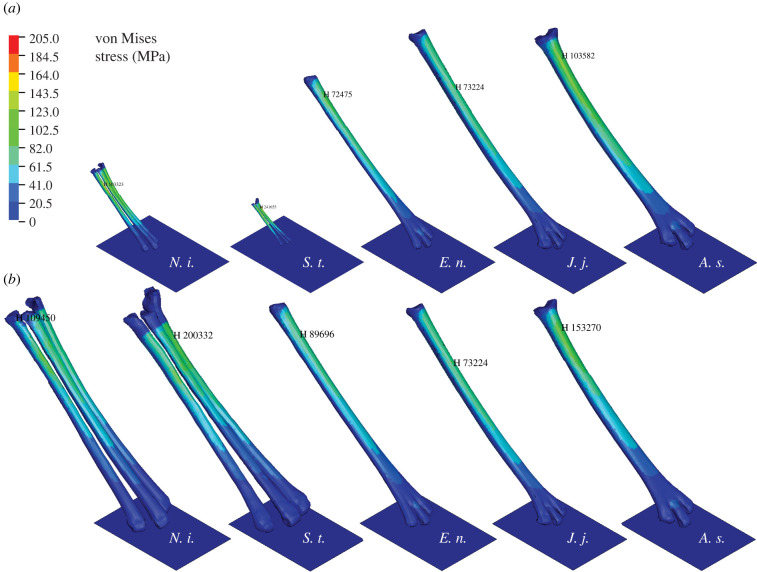
Species models showing von Mises stresses for (*a*) unscaled models and for (*b*) scaled models. Note that the colour scale is the same for all species. The location of maximum von Mises stress is indicated by the element number. (Online version in colour.)

For each model, histograms of the peak stress for each node indicate the von Mises stress concentration and the proportion of nodes above the fracture stress (205 MPa [37], figures 3; electronic supplementary material, S4). As expected for cantilever bending, the dorsal surface of the bones indicated loading in compression (in the *Y* and *Z* axes, specifically) and the plantar surface of the bones indicated loading in tension (see plots of longitudinal stresses in the Deep Blue Data repository). The proximal junction between the metaphysis and the diaphysis consistently developed the highest stresses across all simulations. Human fractures of the central metatarsals generally tend to be evenly spaced between distal and proximal diaphyses [38], so the region of highest stress is probably driven more proximal than expected owing to the way in which the proximal epiphysis was constrained in displacement and rotation. This constraint will also probably increase the maximum von Mises stress values, owing to restricting rotation in the sagittal plane at the ankle joint. Peak stresses occurred in the time interval directly following maximum plate displacement.

The *J. jaculus* model remained unscaled in both simulations and reached a peak ground reaction force of 3.41 N. Peak ground reaction forces for unscaled models reached: *N. insignis* 0.865 N, *Sa. thomasi* 0.284 N, *E. naso* 1.25 N, *A. sibirica* 4.36 N, which varied significantly with body size (linear model, *p* = 0.02, adjusted *R*^2^ = 0.81). Peak ground reaction forces for scaled models reached: *N. insignis* 3.24 N, *Sa. thomasi* 4.43 N, *E. naso* 1.82 N, *A. sibirica* 4.63 N, which did not vary with body size (linear model, *p* = 0.6, adjusted *R*^2^ = −0.19).

### (a) Unscaled model stresses

**Figure 4.**
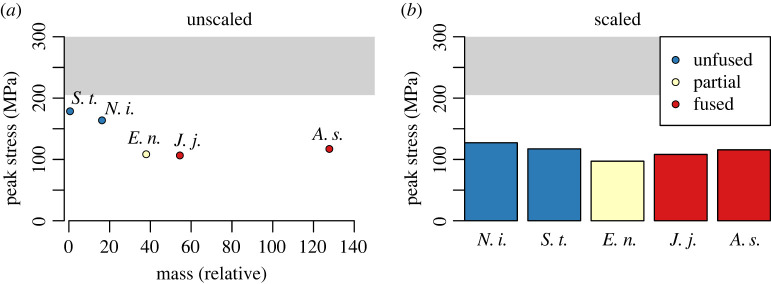
(*a*) Peak von Mises stresses plotted against relative body mass for each unscaled model, and (*b*) each scaled model. Note that the species with the lowest body mass is the obligately bipedal jerboa (*Sa. thomasi*), and the species with the next lowest body mass is the facultatively bipedal *N. insignis*. Colours indicate metatarsal fusion. The grey region represents stresses above the maximum stress for bone as modelled in the simulation (205 MPa), and peak stresses in the region would indicate likely fracture. Relative body mass is calculated from the exponent of phylogenetic principal components analysis 1 (pPC1) scaled to literature body mass values for jerboas (electronic supplementary material, figure S1*a*). (Online version in colour.)

The unscaled models (figure 4*a*), indicate that the fused metatarsals of obligately bipedal jerboas develop lower peak stresses than unfused metatarsals in response to displacement relative to body size. The lowest stresses develop in *J. jaculus* (108.1 MPa), the fully fused obligately bipedal jerboa that served as our reference species. The smallest species, the unfused obligately bipedal pygmy jerboa *Sa. thomasi*, develops the highest peak stresses in response to displacement relative to true body size (180.2 MPa). No species indicated failure by developing stresses higher than 205 MPa.

### (b) Scaled model stresses

In the scaled models (figure 4*b*), the lowest peak stresses develop in the partially fused metatarsals of the obligately bipedal jerboa, *E. naso* (97.1 MPa), scaled to the size of *J. jaculus*. The highest peak stresses develop in the unfused facultatively bipedal rodent, *N. insignis* (127.2 MPa). As in the unscaled models, all of the fused metatarsals develop lower stresses than the unfused metatarsals. The peak stresses do not vary significantly with body size for unscaled models (linear model, *α* = 0.05).

## 4. Discussion

Although biomechanically beneficial traits are not necessarily the result of evolutionary selective pressures, the bipedal locomotion of jerboas requires modifications in hindlimb morphology. For these rodents, feet are integral for escaping predators and for foraging, so failure in the bones of the foot would be detrimental to the animal’s survival [13]. Therefore, we would expect these structures to have a high safety factor, the ratio of the failure stress to the functional stress [16]. While direct measurements of functional stresses are unavailable for many of the animals in this study, the loading conditions are informed by empirical data collected during the locomotion of one species. The results provide valuable context for understanding how fusion and body size affect the ability of a metatarsus to withstand loading conditions that are typically associated with bipedal locomotion.

### (a) Fusion greatly reduces bipedal bone loading

The results from the unscaled simulations do not support hypothesis 1 (less body mass reduces relative bone loading), but the scaled simulations do support hypothesis 2 (fusion reduces the magnitude of stress under similar loading conditions). Although none of the models indicated fracture under the specified modelling conditions, both unscaled and scaled simulations revealed that partially and fully fused metatarsals developed lower stresses than either example of unfused metatarsals.

If hypothesis 1 is not supported by unscaled simulations, how can rodents with unfused metatarsals perform locomotion that develops stresses so close to their ultimate strength? For the facultatively bipedal *N. insignis*, these high stresses may be acceptable because they are so rare. Potential fracture of metatarsal bones is less likely to occur during normal quadrupedal locomotion than during bipedal predator evasion behaviours. However, the obligately bipedal *Sa. thomasi* must encounter the ground reaction forces associated with bipedal locomotion more frequently. One potential explanation is that the dynamics of bipedal locomotion exhibited by pygmy jerboas differ significantly from the locomotion of larger jerboas. Because the kinematics and dynamics used to model each species were not directly measured, scaling the boundary conditions associated with the *J. jaculus* experimental data to the body sizes of each species depends on the assumption that bipedal locomotion is dynamically similar to estimate the peak stresses each metatarsus would develop. Significant differences in gait use and trajectory unpredictability have been observed even between pairs of jerboas with fully fused metatarsals [13,39], but it is unclear whether these arise from significantly different dynamics. For example, differences in locomotor dynamics might result in changing the foot angle at touchdown. Empirical collection of kinematic and dynamic data from jerboas that span the complete range of sizes and metatarsal morphologies is necessary to determine whether bipedal jerboa locomotion can be adjusted to reduce bone loading.

The scaled simulations demonstrate how stresses would develop in metatarsal bones if *J. jaculus* had retained more ancestral morphologies. The unfused metatarsals develop the highest stresses, which supports expectations from both structural and evolutionary perspectives. The fully fused metatarsals developed the second highest stresses, whereas the partially fused metatarsals developed the lowest stresses. From an evolutionary perspective, we expected the fully fused metatarsals to develop the lowest peak stresses in response to the same boundary conditions because these species represent the most recently diverging crown group of bipedal jerboas with the most derived traits. In particular, because the *Allactaga* genus includes the largest species of jerboa (*A. major*, not included in this study), we expected the closely related *A. sibirica* to be the most robust. Instead, an intermediate jerboa, rather than the most derived jerboas, develops the lowest stresses in response to the ground-reaction forces associated with bipedal locomotion.

### (b) Partial fusion

Puzzlingly, *E. naso* is the only species in its genus, and the only species of jerboa known to have partially fused metatarsals. It is important to note that *E. naso* is not an evolutionary transition species between unfused and fully fused jerboas, but it is an extant example of an intermediary morphology between unfused and fully fused metatarsals. Furthermore, *E. naso* closely resembles an early developmental state of the fully fused *J. jaculus* [19]. This begs the question: if partial fusion provides the strongest metatarsus, why do later diverging and larger species evolve fully fused metatarsals? Although peak stresses of the fully fused metatarsals are higher than those of *E. naso*, they did not result in any fractures and their magnitudes were generally lower than those of the unfused metatarsals. There may have been some ‘evolutionary momentum’ associated with fusing metatarsals past the optimal state, perhaps owing to the nature of the developmental mechanism [19]. Because the fully fused state still provides structural reinforcement, this overshoot past the functional optimum may have persisted as a neutral trait with sufficient, if not optimal, fitness [40]. Alternatively, larger jerboas may behaviourally compensate for sub-optimal metatarsal strength by reducing their maximum jump height or peak accelerations. Or, because the more radially symmetric cross-sections of the fully fused metatarsals probably resist forces in multiple directions more consistently, full fusion might reflect more complex ground reaction forces associated with more unpredictable locomotion (electronic supplementary material, figure S3). It is also possible that fully fusing metatarsals reduces the moment of inertia of the limb, which could potentially reduce the energetic cost of redirecting the limb in the swing phase of locomotion [41,42], but experimental data have demonstrated that gait kinematics can change to maintain low energetic cost with limbs that have higher moment of inertia [43,44]. Future studies can incorporate the functional constraints identified by finite-element modelling into phylogenetic models of trait evolution to better understand the processes contributing to overshooting the optimum morphology in crown-group jerboas.

### (c) Finite-element analysis complements comparative methods

The FEA results provide important context for interpreting ancestral state reconstructions of jerboa trait evolution. The ancestral state reconstructions of phylogenetic principal components analysis 1 (pPC1) indicates that the common ancestor of all bipedal jerboas is likely to have been similar in size to the facultatively bipedal *N. insignis*. The results of this study indicate that body size has less of an effect on bone loading than metatarsal fusion, so the moderately sized ancestor would have been biomechanically feasible. However, if hypothesis 1 had been supported, then the combination of a larger body size, bipedal locomotion and unfused metatarsals suggested by the ancestral state reconstruction for the common ancestor of jerboas would have been biomechanically unlikely. Because ancestral state reconstruction consists of weight averaging of extant species, if metatarsal fusion had enabled subsequent jerboas to increase in body size, the miniaturization bottleneck would be statistically impossible to detect using comparative phylogenetic methods alone. Although in this case, the FEA results support the ancestral state reconstructions, we encourage future comparative phylogenetic studies of morphological traits to explicitly assess whether the reconstructed states would be biomechanically feasible.

### (d) Advantages of dynamic finite-element analysis

In comparison to static FEA, dynamic FEA incorporates an acceleration matrix and the mass of the bodies to more accurately model the physics of the system. This is particularly important to include for models of situations in which the load is owing to a rapid collision between bodies, such as foot contact during locomotion, headbutting behaviours [45], and rapid strikes to shatter hard prey [46]. Static FEA is probably sufficient to model behaviours that do not involve rapid decelerations, such as chewing [47], piercing soft tissue [48] and post-collision grappling of horns and antlers [32].

### (e) Future work

This methodology can be used to understand the process of morphological evolution accompanying transitions from quadrupedal to bipedal locomotion in other taxa. Despite similarities to jerboas in body size and ecology, kangaroo rats (Heteromyidae) and Australian hopping mice (Muridae) have no metatarsal fusion [49,50]. These convergent rodents may have undergone a distinct set of morphological changes that enabled the transition to bipedal locomotion. Indeed, the behavioural and biomechanical differences among these groups are so significant that they might not be considered fully convergent [51]. For example, Australian hopping mice are only facultatively bipedal [52], more equivalent to *N. insignis*. Additionally, there are significant differences in evolutionary history among the three groups. Jerboas also have the most extreme morphology and the most extreme locomotion of all bipedal hopping rodents [53]. A modelling approach can be used to compare the lineage-specific effects on trait evolution in response to similar selective pressures across these convergent groups.

Similar methods can probably be used to accurately analyse more complex biological structures, including those with joints involving multiple materials or complex dynamics between multiple structures [54], in complex loading conditions. With these more complex models, it would be possible to simulate how an articulated fossil foot, such as those found in early hominids, would change shape and develop stresses during different modes of locomotion. Because of the increased complexity required for such simulations, using computing clusters can greatly enhance our ability to model joints during collisions.

## 5. Conclusion

Finite-element modelling of dipodoid metatarsal loading revealed how metatarsal fusion helps jerboas compensate for the higher hind limb ground reaction forces associated with bipedal locomotion in this clade. Jerboas evolved metatarsal fusion, which provides structural reinforcement and releases the evolutionary constraint on body size. However, full fusion in crown-group jerboas may be an example of evolutionary overshoot past the mechanical optimum of partial fusion or an adaptation to reinforce the metatarsals in response to loading in multiple directions. Modelling biomechanical interactions can provide important context for the statistical reconstruction of extinct ancestral morphologies. Our results support the hypothesis that the common ancestor of all bipedal jerboas could have had a moderate body size and unfused metatarsals. Investigating multiple functional mechanisms within the same clade may be a useful approach for examining the evolution of other biomechanical transitions.

## Data Availability

μCT scans, morphological measurements and modelling files are available at the DeepBlue Data Repository https://doi.org/10.7302/cdk6-vx27 [55]. The data are provided in the electronic supplementary material [56].
